# 

**Published:** 1842-11

**Authors:** 


### WESTERN JOURNAL

OF

### MEDICINE AND SURGERY.

### EDITORS.

---

DANIEL DRAKE, M. D.,

PROFESSOR OF PATHOLOGICAL ANATOMY AND CLINICAL MEDICINE IN THE MEDICAL INSTITUTE OF LOUISVILLE:

LUNSFORD P. YANDELL, M. D.,

PROFESSOR OF CHEMISTRY AND PHARMACY IN THE SAME:

THOMAS W. COLESCOTT, M. D.

### RECEIPTS OF THE MEDICAL JOURNAL.

FOR THE MONTHS OF SEPTEMBER AND OCTOBER.

| | | | |
|---|---|---|---|
| Dr. J. Kirkwood, New Madrid, Mo., | - | - | $5 00 |
| A. C. Dement, Mt. Pulaski, Ill., - | - | - | 5 00 |
| Stark County Medical Society, Canal Fulton, O., | | - | 5 00 |
| Dr. W. P. Jones, Bowling Green, Ky., | - | - | 5 00 |
| H. Day, Pendleton, Ohio, - | - | - | 5 00 |
| L. Brooks, Harrisonville, Missouri, | - | - | 5 00 |
| —— Bearce, Decatur, Ohio, - | - | - | 5 00 |
| J. Moore, Unionville, Tennessee, - | - | - | 5 00 |
| J. P. Henderson, Newville, Ohio, | - | - | 5 00 |
| Drs. Porter & Whitten, Rockford, Indiana, | - | - | 5 00 |
| Dr. Means, Preston, Mississippi, - | - | - | 1 00 |
| W. M. Stansberry, Carrollton, Mississippi, | | - | 5 00 |
| S. Borland, Memphis, Tennessee, | - | - | 5 00 |
| —— Patrick, Kanawha, Virginia, | - | - | 13 00 |
| W. O. Edwards, Portersville, Tennessee, - | | - | 5 00 |
| F. McFarland, Alexandria, Louisiana, | - | - | 5 00 |

The Western Journal of Medicine and Surgery is published monthly by the undersigned, at the corner of Main and Fifth streets, Louisville, at $5 per annum, payable in advance. Each number contains from 80 to 84 pages making two volumes in the year of about 500 pages.

Contributions to its pages by the Physicians of the Valley of the Mississippi, addressed to the Editors, are respectfully solicited.

Letters on business, to be addressed, postage paid, to the publishers.

☞Postmasters, by the regulations of the Postoffice Department, will frank letters containing subscription money, and all remittances so franked are at the risk of the publishers.

*August*, 1842. PRENTICE & WEISSINGER.

### DELINQUENT LIST.

We have at length completed the list of such as have never paid any thing on this Journal—that is, of all such out of Kentucky. These patrons of learning now have the pleasure of contemplating their names in print, and at the same time an epitome of their character for liberality, honor and honesty, and if they will just let this cover get abroad in their neighborhoods, their neighbors will know them as well as we do. Whenever, gentlemen, you are tired of contemplating the picture, just send on the funds, and we will take the mirror down. The Post-master will frank.

TENNESSEE.

J. Drake, South Carroll
J. J. Gibson, Hampshire
Wilson & Bearden, Petersburgh
L. M. N. Cook, Silver Spring
T. J. Walton, Mulloy's
S. B. Robinson, Versailles
J. M. Dean, Lynchburg
A. F. Clement, Lexington
W. H. Warren, Lexington
W. T. Eatherty, Rural Hill
J. A. Lecky, Ripley
S. Richardson, Rutherford.
W. D. Lee, Rutherford.
W. J. J. Morrow, Chattawogga
J. W. Koen, Colliersville
J. M. M. Cornelius, Germantown
Robinson & Hagan, Carthage
J. B. Copeland, Nolandsville
R. Forbis, Elkton
M. W. O'Brien, Springfield.
W. J. C. Rogers, Murfreesboro'

NEW YORK.

Wm. Welch, Penfield
G. Standing, Utica

ARKANSAS.

W. A. Threlkeld, Helena
J. H. McCarty, Ozark
Dr. Beach, Lawrenceville
J. Eaton, Pine Bluff

LOUISIANA.

B. B. Smith, Shreveport
J. B. Henderson, New Orleans.
J. Davis, Millekin's Bend
T. Ingersoll, Cherryville
E. Ragan, Columbia

ALABAMA.

P. L. Gilbert, Isny, Washington County.
R. L. Graves, Leesville
D. W. Ford, Meridianville
E. M. Hussey, Waterloo
W. R. Davie, Huntsville
W. F. Johnson, Benton
J. Durno, Mobile, or Alton, Ill.
J. T. Reese, Syllacogga.

MISSISSIPPI.

Harris & McLaen, Wahallock
E. F. Bouchelle, Columbus
S. D. Kennedy, Holmesville
D. B. Nailer, Vicksburgh
—— Hogan, Panola
A. P. Reed, Fort Adams
J. A. Hodges, Garlandsville
O. S. Echols, Hamilton
L. Shelby, Warrenton

ILLINOIS.

O. Abbey, Quincy
W. H. Efner, Albany
J. W. Chenoweth, Auburn
C. L. Duncan, Marshall
J. R. Colwell, Farmington
J. Kirkpatrick, Fox P. O.
W. Allison, Paradise
W. Patton, Oakland
T. J. Beckwell, Rushville
J. Jayne, Carlinsville
B. F. Edwards, Alton
C. F. Falley, York
H. Symmes, Equality
W. O. Chamberlain, Princeton
Wood & Goodwin, Holland's Grove
F. Canterbury, Cole's C. H.
H. P. Griswold, Plymouth
J. M. Metcalf, Winchester
W. Kellar, Decatur

INDIANA.

B. F. Russell, New Washington
J. H. McNutt, Annapolis
D. Long, Blackford
D. Hoover, Wolf Lake
J. L. Plaff, Westfield
F. C. Gale, Vevay
N. B. Odelle, Lafayette
J. M. Bell, Dublin
P. Q. Stryker, Rockville.
W. H. Martin, Rushville
C. West, Richmond
L. Edwards, Greenfield
J. A. Pomeroy, Roseville
E. H. Bruce, Peru
S. P. Wort, Brownstown.
J. Prosser, Orleans
H. Single, Orleans
T. Losey, Laporte
W. Simpkins, Lebanon
W. M. Gentry, Frankfort
W. V. Snyder, Frankfort
R. Hobson, New Harmony
J. H. Herryman, Bowling Green
D. Todd, Monticello
A. Poteet, Monticello
C. Wallace, Bellville
S. T. Clark, Russelville
J. W. Porter, Greenville
J. C. Clarke, Greenville
T. Sturges, Fort Wayne
A. D. Sweet, Franklin
E. S. Blue, Leesburgh
J. Darr, New Castle
J. V. Wayman, New Castle
J. W. Crooks, Rockport
J. W. French, Troy
A. M. Murphy, Bloomington
I. N. Griffith, Jeffersonville
D. C. McGee, Mount Pleasant
J. McCallen, McAllen's Cross Roads
J. S. Deputy, Madison
—— Tilton, Madison
S. Wyatt, Lexington
W. W. Goodwin, Charlestown
G. R. McCoy, Charlestown

MICHIGAN.

G. W. Gorham, Jackson
R. Chase, New Arbor
E. W. Cowles, Brest
J. Allen, La Grange

OHIO.

J. Stone, Sharon
R. G. McLaen, Rokeby
O. H. P. Baer, Union
H. L. Thrall, Gambier
G. F. Mitchell, Olivesburgh
J. Clark, Anderson's Store
E. P. Dameron, Point Pleasant
G. E. Conant, Huntington
—— Stone, Marietta
S. J. Spees, Lynchburgh
D. Cox, New Paris
S. Hitchcock, Princeton
A G. McFarland, Christiansburgh
C. G. Espick, Germantown
C. Thiel, Amanda
J. Holland, Logan
R. D. Lilley, Hillsboro'
J. C. Kennedy, Felicity
—— Bell, Belbrook
R. & J. B. Thompson, Columbus
J. N. Green, Bainbridge
R. Hewitt, Port Washington
L. N. Wilcoxon, Moscow

PENNSYLVANIA.

J. R. Wickey, West Middlesex
A. C. Brainard, Pymatuning
J. Thompson, New Geneva
T. Swaby, Bloomingsburgh

VIRGINIA.

Cambell & Wheeler, Wellsburgh
T. Goode, P. M., Hot Springs

GEORGIA.

T. F. Gibbs, Ruckersville
J. H. M. Barrett, Ruckersville
W. Wells, Rockbridge
R. F. Ogilby, Rome
J. H. Ferrensides, Drayton
H. L. Burt, Mt. Zion

MISSOURI.

P. Conduitte, Monticello
Isaac Lea, Chantilly
P. M. Dickinson, Cape Girardeau
L. Lemis, Osceola
M. B. McElroy, Warren
J. S. Bledsoe, Matthew's Prairie
C. W. Baker, New Franklin

WISCONSIN TERRITORY.

Post Library, Fort Winnebago

NORTH CAROLINA.

J. M. Happolett, Charlotte

